# Matrix Metalloproteinase-10 Is Required for Lung Cancer Stem Cell Maintenance, Tumor Initiation and Metastatic Potential

**DOI:** 10.1371/journal.pone.0035040

**Published:** 2012-04-24

**Authors:** Verline Justilien, Roderick P. Regala, I-Chu Tseng, Michael P. Walsh, Jyotica Batra, Evette S. Radisky, Nicole R. Murray, Alan P. Fields

**Affiliations:** Department of Cancer Biology, Mayo Clinic College of Medicine, Jacksonville, Florida, United States of America; The University of Texas M.D Anderson Cancer Center, United States of America

## Abstract

Matrix metalloproteinases (Mmps) stimulate tumor invasion and metastasis by degrading the extracellular matrix. Here we reveal an unexpected role for Mmp10 (stromelysin 2) in the maintenance and tumorigenicity of mouse lung cancer stem-like cells (CSC). Mmp10 is highly expressed in oncosphere cultures enriched in CSCs and RNAi-mediated knockdown of *Mmp10* leads to a loss of stem cell marker gene expression and inhibition of oncosphere growth, clonal expansion, and transformed growth *in vitro*. Interestingly, clonal expansion of *Mmp10* deficient oncospheres can be restored by addition of exogenous Mmp10 protein to the culture medium, demonstrating a direct role for Mmp10 in the proliferation of these cells. Oncospheres exhibit enhanced tumor-initiating and metastatic activity when injected orthotopically into syngeneic mice, whereas Mmp10-deficient cultures show a severe defect in tumor initiation. Conversely, oncospheres implanted into syngeneic non-transgenic or *Mmp10* ^−/−^ mice show no significant difference in tumor initiation, growth or metastasis, demonstrating the importance of *Mmp10* produced by cancer cells rather than the tumor microenvironment in lung tumor initiation and maintenance. Analysis of gene expression data from human cancers reveals a strong positive correlation between tumor Mmp10 expression and metastatic behavior in many human tumor types. Thus, *Mmp10* is required for maintenance of a highly tumorigenic, cancer-initiating, metastatic stem-like cell population in lung cancer. Our data demonstrate for the first time that *Mmp10* is a critical lung cancer stem cell gene and novel therapeutic target for lung cancer stem cells.

## Introduction

Non-small cell lung cancer (NSCLC) is the leading cause of cancer death in the United States [1]. Outcome for NSCLC patients remains poor, underscoring the need to identify more effective means of prevention, diagnosis, and treatment. Tumors contain subpopulations of cells exhibiting stem-like characteristics that drive pathogenesis [2], [3], [4]. These cancer-initiating or cancer stem cells (CSCs) exhibit self-renewal, tumor-initiating activity, clonal expansion as “oncospheres”, the ability to support tumor maintenance and metastasis, and to differentiate into the transiently-amplifying cells comprising the bulk tumor [2], [4], [5], [6]. CSCs share molecular features with embryonic stem cells, including expression of aldehyde dehydrogenase (Aldh) [7], and stem cell markers such as CD133 [8], [9], Nanog [10], Oct4 [10], Sox2 [11] and Notch receptors [12]. CSCs have been described in many forms of human cancer including lung cancers [9], [13]. The central role of CSCs in tumorigenesis indicates that these cells must be targeted therapeutically to achieve effective cancer treatment.

Mmps have been implicated in lung tumor proliferation, invasion, and metastasis [14]. The stromelysin subfamily [stromelysin 1 (Mmp3), 2 (Mmp10) and 3 (Mmp11)] is often overexpressed in NSCLC [15], . Interestingly, Mmp10 is highly expressed in NSCLC tumors but not tumor-associated stromal cells, whereas Mmp3 and Mmp11 are expressed predominantly in stroma [17], . We recently demonstrated that Mmp10 is required for transformed growth and invasion of human NSCLC cells *in vitro* [19], is induced in bronchio-alveolar stem cells (BASCs) transformed by oncogenic *Kras* [20], and promotes *Kras*-mediated lung tumorigenesis in vivo [21]. *Mmp10*-deficient mice exhibit a block in *Kras*-and urethane-induced tumor formation, an effect that correlates with an inability of Mmp10-deficient BASCs to expand and undergo transformation in response to urethane or *Kras* [21]. Thus, Mmp10 mediates tumor initiation through control of tumor-initiating cell (BASC) expansion.

Here, we assess the role of Mmp10 in the maintenance and tumorigenic potential of fully-transformed mouse lung CSCs. Cultures enriched in CSCs from mouse lung adenocarcinoma cells express elevated Mmp10, Nanog, Aldh1, CD133, Notch3, Notch4, Hey1 and Hey2. These cultures exhibit increased anchorage-independent growth in vitro, and enhanced tumor initiation, growth and metastatic spread as orthotopic tumors in syngeneic mice. These stem-like properties require expression of Mmp10 in tumor cells but not in tumor-associated stroma. Our results indicate that Mmp10 is an attractive candidate for the development of mechanism-based therapy targeting highly tumorigenic lung CSCs.

## Results

### Mouse lung oncospheres express elevated Mmp10 and Mmp10-dependent stem-like properties

**Figure 1:**
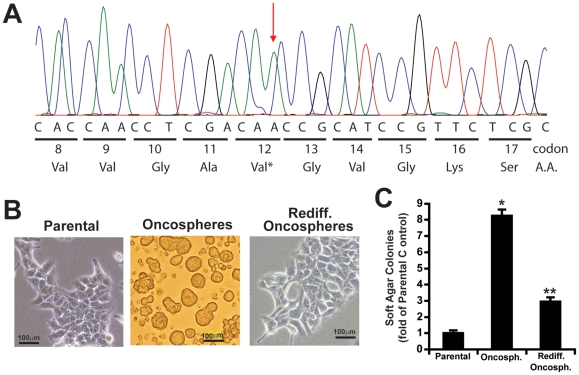
Enrichment of CMT167 cancer stem cells. **A**) CMT167 cells harbor an activating *Kras^G12V^* mutation. DNA sequence chromatogram of *Kras* allele in CMT167 cells reveals a codon 12 mutation from glycine to valine (*Kras^G12V^*) (**red arrow**). **B**) Phase contrast photomicrographs of parental adherent CMT167 cells (***left panel***), CMT167 oncospheres in stem cell culture (***middle panel***) and redifferentiated oncosphere cells after return to adherent culture (***right panel***). **C**) CMT167 oncosphere cultures exhibit enhanced anchorage-independent growth. Mean fold-change from CMT167 parental cells +/−SEM. n = 5, *p<0.00001; **p<0.00001.

*Mmp10* is highly induced in tumor-initiating lung bronchio-alveolar stem cells (BASCs) upon activation of oncogenic *Kras* [20]. Furthermore, *Mmp10*-deficient mice exhibit decreased *Kras*-mediated lung tumor formation and a loss of *Kras*-mediated BASC expansion in vitro and in vivo [21]. To directly explore the importance of Mmp10 in lung CSC biology, we analyzed mouse CMT167 cells, a highly tumorigenic, metastatic cell line derived from a spontaneous alveolar lung adenocarcinoma in a C57BL/6 mouse [22], [23]. DNA sequencing revealed that CMT167 cells harbor an activating *Kras^G12V^* mutation (**Figure 1A**), making it an ideal cellular model to study Mmp10 in mutant *Kras* lung CSCs. In contrast to CMT167 cells grown in adherent culture (**Figure 1B** **, left**), cells grown in defined stem cell medium in low-adherence plates grow as oncospheres (**Figure 1B** **, middle**) reminiscent of CSC cultures from human lung cancer cell lines [13]. These oncospheres redifferentiate when returned to adherent culture, exhibiting morphology comparable to parental cells (**Figure 1B** **, right**). CMT167 oncosphere cultures exhibit a ∼8-fold increase in anchorage-independent colony formation compared to parental cells (**Figure 1C**), consistent with the enhanced tumorigenic properties of CSCs [13]. Enhanced colony formation is largely lost when these cells are allowed to redifferentiate by returning them to adherent culture (**Figure 1C**). Similar results were obtained using a second mouse lung adenocarcinoma cell line, the Lewis Lung Carcinoma (LLC) (**Figure S1A** and **B**). LLC cells grown as oncospheres in stem cell culture exhibit enhanced anchorage-independent colony formation, but lose these properties when returned to adherent culture. These results demonstrate that our oncosphere cultures are enriched in cancer stem-like cells.

**Figure 2:**
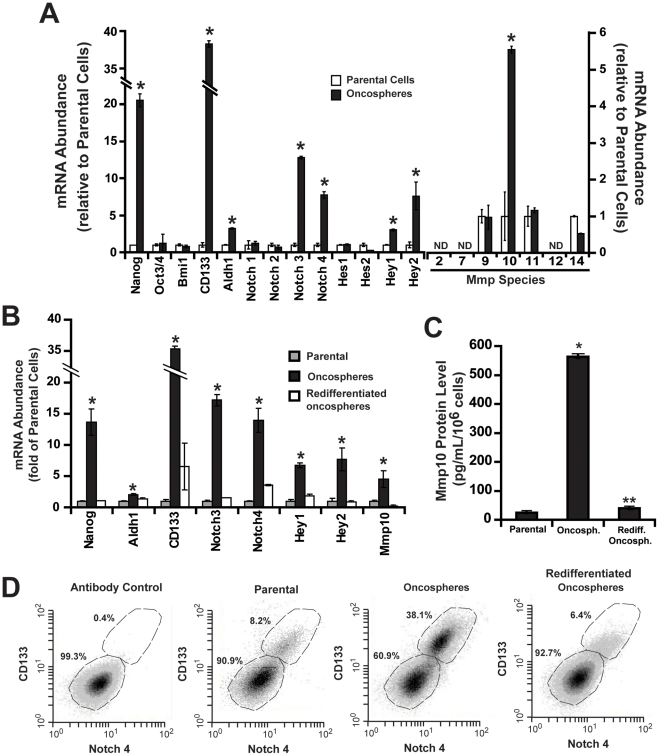
Molecular and functional characterization of CMT167 oncosphere cultures. **A**) Expression of cancer stem cell markers in CMT167 parental and oncosphere cultures. QPCR of cancer stem cell-associated genes; *p<0.05. **B**) QPCR for stem cell markers in parental, oncosphere and redifferentiated oncosphere cultures. Fold of parental cells +/−SEM, n = 3; *p<0.05. **C**) CMT167 oncosphere cultures secrete elevated Mmp10 protein. ELISA of culture supernatants from parental, oncosphere and redifferentiated CMT167 oncosphere cultures. Mean +/− SEM. n = 3, *p<1×10^−8^ parental vs. oncospheres; **p<2×10^−8^ oncospheres vs. redifferentiated oncospheres. **D**) Flow cytometry of CMT167 parental, oncosphere and redifferentiated oncosphere cultures for CD133 and Notch4.

To further characterize our oncosphere cultures, we assessed them for expression of well-characterized stem cell-associated genes. QPCR revealed that CMT167 oncosphere cultures express elevated mRNA for many genes associated with the stem cell phenotype [24] including Nanog, Aldh1, CD133, Notch3, Notch4, Hey1 and Hey2 (**Figure 2A**). Strikingly, these cultures also express elevated Mmp10 mRNA (**Figure 2A**), but no increase in Mmp2, Mmp7, Mmp9, Mmp11, Mmp12 or Mmp14, other Mmp species commonly linked to lung cancer (**Figure 2A**). As expected, oncosphere cultures exhibit a loss of stem cell markers and Mmp10 expression when allowed to redifferentiate in adherent culture (**Figure 2B**). A similar pattern of stem cell marker expression was observed in oncosphere cultures from LLC cells, including a dramatic increase in MMP10, indicating that these changes are not unique to CMT167 oncospheres (**Figure S1C**). CMT167 oncosphere cultures secrete elevated Mmp10 protein into the medium compared to parental or redifferentiated cultures (**Figure 2C**), demonstrating the functional significance of elevated Mmp10 expression in these cultures. CD133 and Notch4 are two highly selective surface markers expressed on other lung CSCs [9], . Therefore, we assessed the expression of these markers in CMT167 parental, oncosphere and redifferentiated oncosphere cultures by flow cytometry (**Figure 2D**). Parental cell cultures contain ∼8% CD133^+^/Notch4^+^ cells. In contrast, oncosphere cultures contain ∼38% CD133^+^/Notch4^+^ cells and redifferentiated cultures ∼6% CD133^+^/Notch4^+^ cells. These results are consistent with the prevalence of CSCs in other lung cancer cell lines [9], , indicating that these are selective markers for CSC-enriched cultures.

**Figure 3:**
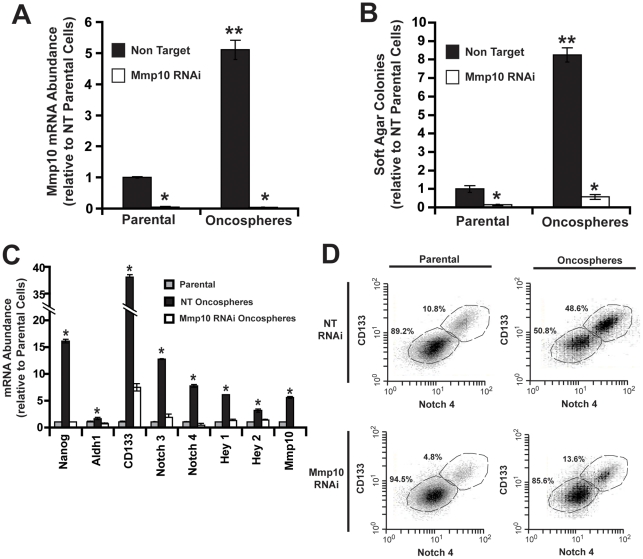
Mmp10 is critical for maintenance and growth of CMT167 oncosphere cultures. **A**) QPCR showing RNAi-mediated knockdown of Mmp10 in parental CMT167 and oncosphere cultures. Relative to NT parental cells; Mean ±s.d., n = 3. *p<0.0002 and **p = 0.01 vs. NT parental control. **B**) Effect of Mmp10 RNAi on anchorage-independent growth of parental and CMT167 oncosphere cultures. Relative to NT parental cells +/− SEM, n = 5; *p<0.05 vs, NT; **p<0.05 vs. NT parental. **C**) QPCR for stem cell markers in NT parental, and NT and Mmp10 RNAi CMT167 oncosphere cells. Fold of NT parental +/−SEM, n = 3; *p<0.05. **D**) Flow cytometry of NT and Mmp10 RNAi parental and oncosphere cells for CD133 and Notch4.

Since oncospheres express elevated Mmp10, we next assessed whether Mmp10 is important for their maintenance and growth. Three RNAi lentiviruses targeting mouse Mmp10 RNA were identified that inhibit Mmp10 expression (**Figure S2A** and **B**). Each Mmp10 knockdown virus caused a commensurate inhibition of anchorage-independent growth compared to non-target (NT) RNAi control cells (**Figure S2C)**. The most effective Mmp10 RNAi construct (#3) was used to elicit efficient knockdown of Mmp10 mRNA in both parental and oncosphere cultures (**Figure 3A**). Mmp10 knockdown caused an inhibition of transformed growth (**Figure 3B**), and a loss in expression of Nanog, Aldh1, Cd133, Notch3 and 4, Hey1 and 2, and Mmp10 (**Figure 3C**). Similar results were obtained in LLC oncosphere cultures in which MMP10 was knocked down by RNAi (**Figure S3A–C**). Mmp10 RNAi oncosphere cultures also exhibited a decrease in CD133^+^/Notch4^+^ cells compared to NT RNAi oncosphere cultures (**Figure 3D**). Interestingly, Mmp10 knockdown also caused a decrease in CD133^+^/Notch4^+^ cells in parental cultures (from ∼11% to ∼5%) (**Figure 3D**), indicating that Mmp10 is important for the maintenance of a CSC population within parental cultures.

**Figure 4:**
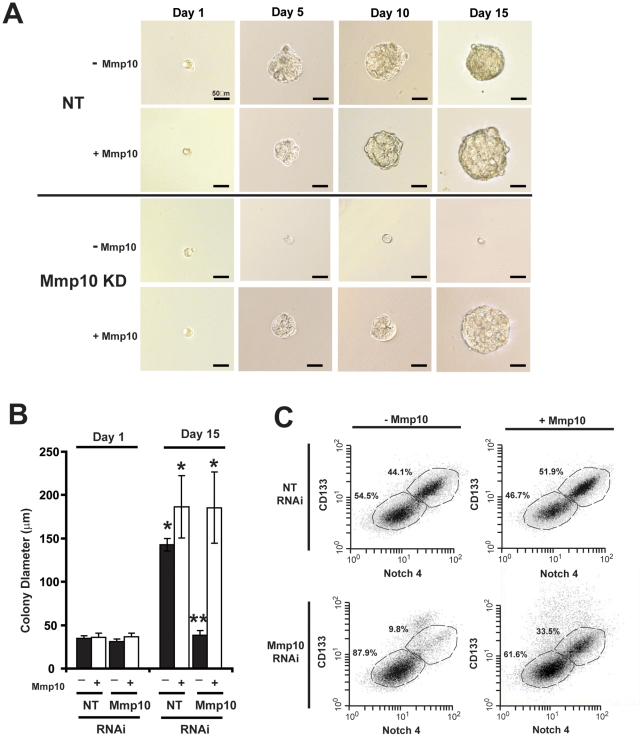
Mmp10 is required for clonal expansion of CMT167 oncospheres. **A**) Photomicrographs of clonal expansion of single NT and Mmp10 RNAi CMT167 oncosphere cells in the presence or absence or recombinant Mmp10. **B**) Colony diameter derived from single CMT167 NT and Mmp10 RNAi cells. Mean +/− SEM; n = 10, *p<0.05 vs. day 1 each treatment; **p<0.05 vs. day 15 NT RNAi without Mmp10. **C)** Flow cytometry of NT and Mmp10 RNAi CMT167 oncosphere cultures +/−Mmp10 for CD133 and Notch4.

We next determined whether Mmp10 is important for clonal expansion of oncospheres, a well-defined characteristic of CSCs. Single Mmp10 RNAi- and NT RNAi oncosphere cells were plated in individual wells of low-adherent tissue culture plates and allowed to clonally expand. Virtually all NT RNAi cells expand into large oncospheres over a 15 day period (**Figure 4A** **, upper**), whereas the majority of Mmp10 RNAi cells remain as single cells (**Figure 4A** **, lower**). Similar results were obtained in LLC oncosphere cultures (**Figure S3D**). The decreased clonal expansion of CMT167 oncospheres was due to genetic loss of Mmp10 since addition of recombinant Mmp10 to the culture medium restored clonal expansion of Mmp10 RNAi cells (**Figure 4A** **, lower**). Quantitation confirmed that NT RNAi oncospheres grow significantly larger than Mmp10 RNAi oncospheres, and addition of recombinant Mmp10 restores growth of Mmp10 RNAi cells to that comparable to NT RNAi cells while having no significant effect on NT RNAi cells (**Figure 4B**). Exogenous Mmp10 also led to re-expression of CD133 and Notch4 on Mmp10 RNAi cells (**Figure 4C**), and re-expression of stem cell markers (Nanog, Aldh1, Cd133, Notch3, Notch4 and Hey2) (**Figure S4A**). The decreased clonal growth of Mmp10 RNAi oncospheres is not due to a loss of cell viability, since these cells proliferate at a rate indistinguishable from NT RNAi oncospheres when placed back into adherent culture (**Figure S4B**).

### Oncosphere cultures exhibit Mmp10-dependent tumor-initiating activity *in vivo*

**Figure 5:**
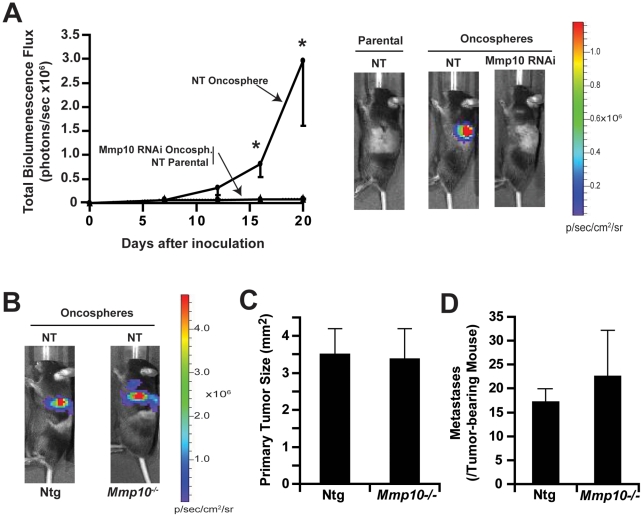
Tumor initiating activity of CMT167 oncosphere cultures is Mmp10 dependent. **A**, Orthotopic tumor growth after injection of 1,000 cells from NT adherent, NT RNAi oncosphere or Mmp10 RNAi oncosphere cultures was monitored by bioluminescence at the indicated time points; *p<0.05 (***left panel***). Representative lateral views of bioluminescent images of mice bearing lung orthotopic tumors (***right panel***). Mmp10 function in CMT167 oncosphere tumors is cell autonomous. **B**) Representative bioluminescent images of NT RNAi CMT167 oncosphere orthotopic tumors implanted into Ntg and *Mmp10* ^−/−^ recipient mice. Analysis of **C**) tumor size and **D**) metastases in Ntg or *Mmp10* ^−/−^ mice. **C–D**, n = 10, Mean+/− SEM.

We next evaluated the tumor-initiating activity of oncosphere and parental cultures by orthotopic injection into the left lobe of the lung of syngeneic mice. Initial experiments determined that injection of 100,000 parental CMT167/luc cells was required to establish orthotopic tumors whereas injection of as few as 1,000 NT RNAi CMT167/luc cells from oncosphere cultures routinely gave tumors. Injection of 1,000 NT RNAi CMT167/luc oncosphere cells led to large tumors whereas injection of 1,000 parental NT RNAi CMT167/luc cells or Mmp10 RNAi CMT167/luc oncosphere cells failed to produce large tumors as determined by live imaging of bioluminescence (**Figure 5A** **)**. Pathological examination of lung tissues at the time of sacrifice demonstrated that tumor take was 100% for NT RNAi CMT167/luc oncosphere cells (9/9) with an average tumor size of 3.63 +/−.35 mm^2^, but only 45% tumor take for parental NT RNAi CMT167/luc cells (5/11) with an average tumor size of 0.42 +/−.06 mm^2^, and 27% tumor take for Mmp10 RNAi CMT167/luc oncosphere cells (3/11) with an average tumor size of 0.36 +/−.09 mm^2^. These data validate our in vivo bioluminescence measurements and demonstrate that Mmp10 plays a critical role in the tumor-initiating activity and growth of CMT167/luc oncosphere cells. Interestingly, Mmp10 knockdown in parental cells also leads to a loss of tumor formation in vivo (**Figure S5A**), indicating that Mmp10 is important for the tumor-initiating activity of a CSC-like cell population present in parental cell cultures. NT RNAi CMT167/luc oncosphere cultures also generated numerous metastases to the right lobes of the lung whereas parental NT RNAi CMT167/luc cell cultures exhibited far fewer metastases and Mmp10 RNAi CMT167/luc oncosphere cultures fewer still (**Figure S5B**), suggesting a role for Mmp10 in metastatic potential, a property ascribed to lung CSCs. Thus, Mmp10 is important for the enhanced tumorigenic potential observed in oncosphere cultures.

### Tumorigenicity is independent of Mmp10 in the tumor microenvironment

The effects of Mmp10 in CMT167 cells *in vitro* are cell autonomous. However, we cannot rule out a role for Mmp10 produced by tumor-associated epithelial, stromal and/or immune cells in our orthotopic tumor studies. To address this issue, we injected 1,000 cells from NT RNAi CMT167/luc oncosphere cultures into either Ntg or *Mmp10* ^−/−^ mice. No significant differences in tumor growth (**Figure 5B**), primary tumor size (**Figure 5C**) or metastases (**Figure 5D**) were seen in Ntg and *Mmp10* ^−/−^ recipient mice, indicating that Mmp10 expressed by oncospheres, but not from Mmp10 produced by other tumor-associated cells, is critical for tumor formation. We previously demonstrated that Mmp10 is expressed in vanishingly low levels in the lung epithelium, but is highly induced in BASCs upon activation of oncogenic *Kras* [20]. Thus, tumor cells are the major source of Mmp10 in the lungs of tumor-bearing mice.

### Oncosphere cultures differentiate into bulk tumor cells after orthotopic injection

**Figure 6:**
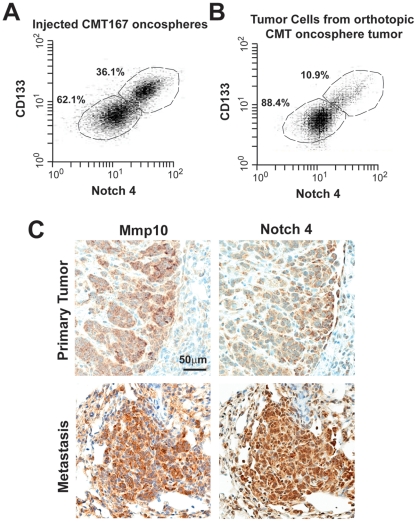
Mmp10 and Notch4 expression in primary and metastatic lung tumors. Flow cytometric analysis for CD133 and Notch4 expression in CMT167 oncospheres prior to injection orthotopically into syngeneic mice (**A**) and CMT167 cells isolated from orthotopic tumors (**B**). **C)** Mmp10 and Notch4 staining in a primary CMT167 oncosphere tumor (***top panels***) and metastatic lesion (***bottom panels***).

CSCs initiate and maintain tumors by maintaining a CSC population and differentiating into transiently-amplifying cells that make up the bulk tumor. To assess the fate of CMT167 oncospheres after orthotropic tumor formation, we isolated tumor cells from orthotopic tumors derived from these cultures and analyzed them for CD133^+^/Notch4^+^ cells (**Figure 6**). At the time of injection, the oncosphere cultures showed significant enrichment in CD133^+^/Notch4^+^ cells (∼36%) (**Figure 6A**). In contrast, tumor cells isolated from orthotopic tumors consisted of ∼11% CD133^+^/Notch4^+^ cells, consistent with parental CMT167 cells (**Figure 6B**). These data indicate that oncosphere cultures propagate a population of CD133^+^/Notch4^+^ stem-like cells, and also differentiate into transiently-amplifying tumor cells in vivo. We also explored the distribution of CMT167 oncosphere cells in orthotopic tumors by immunohistochemistry for Mmp10 and Notch4 (**Figure 6C**). Primary tumors derived from oncospheres exhibited slightly elevated Mmp10 staining with darker Mmp10 staining in areas where the tumor interacts with surrounding stroma. Tumor-associated stroma and normal lung epithelium stain negative for Mmp10, consistent with the paucity of Mmp10 expression in normal lung epithelium [20]. Notch4 staining exhibited a similar pattern, indicating co-expression of Mmp10 and Notch4 in tumor cells at the interface of the tumor and surrounding stroma (**Figure 6C** **, upper**).

### Mmp10 expression is associated with metastasis of lung cancer cells

Recent evidence indicates that CSCs reside in pre-metastatic niches responsible for metastatic tumor spread. Given the association of stemness and metastatic potential, we assessed the expression of Mmp10 and Notch4 in metastatic lesions from tumors derived from oncospheres. Interestingly, both Mmp10 and Notch4 staining was intense and uniform in metastatic lesions (**Figure 6C** **, lower**), indicating that these lesions are enriched in Mmp10-Notch4 double positive CSCs. A similar association of Mmp10 expression with metastatic lesions in human cancers was observed when we analyzed publicly-available gene expression datasets of human tumors using the NextBio bioinformatics tool. Analysis revealed a strong positive correlation between Mmp10 expression and metastatic potential in human NSCLC, colorectal cancer, melanoma, breast cancer, renal cell carcinoma and prostate cancer (**Table 1**). Thus, Mmp10 appears to play a widespread role in human malignancy.

**Table 1:** Mmp10 expression correlates with metastasis in human cancer types.

Tumor Type-Comparison	Fold Change	P value	Reference
Colorectal Cancer-recurrent metastatic vs non metastatic cancer	2.29	0.042	[39]
Melanoma metastasis – proliferative molecular subtype vs pigmentation	9.89	0.0012	[40]
Primary breast tumor – relapse metastasis in lung vs local relapse	2.04	0.0467	[41]
NSCLC from patients with metastasis status M1 vs M0	4.4	0.025	[30]
Renal Cell Carcinoma metastasis vs primary tumor	1.46	0.0403	[42]
Colorectal Cancer -liver metastasis vs primary tumor	3.71	3.00E-15	[43]
Renal Cell Carcinoma – metastasis to lymph nodes/mediastimum vs no metastasis	2	6.00E-04	*GSE22541
Prostate Cancer -peritoneal lymph node metastasis vs primary tumor	1.8	7.20E-06	[44]
Prostate Cancer -liver metastasis vs primary tumor	1.79	0.0072	[44]
Prostate Cancer -metastatic tumor vs primary tumor	1.64	5.90E-06	[44]
Prostate Cancer -tracheal lymph node metastasis vs primary tumor	1.48	6.00E-04	[44]

## Discussion

A small subpopulation of self-renewing stem-like cells may be responsible for the initiation, maintenance, progression and metastatic spread of tumors. However, relatively little is known about the molecular mechanisms involved in CSC maintenance. CSCs can escape conventional chemotherapy, due to their intrinsic drug resistance and/or relative quiescence, resulting in disease recurrence and poor patient outcome. Therefore, therapeutic strategies aimed at eradicating CSCs may improve clinical outcome of cancer patients.

Though Mmps have long been implicated in tumor progression and metastasis, we recently demonstrated that Mmp10 is overexpressed in human NSCLC and *Kras*-transformed BASC lung cancer-initiating cells [19], [20], and that Mmp10 is required for transformed growth and invasion of human NSCLC cells *in vitro* [19]. Furthermore, Mmp10^−/−^ mice exhibit a significant decrease in urethane- and *Kras*-induced lung tumorigenesis due to a defect of Mmp10-deficient BASCs to expand in response to oncogenic *Kras* [21]. Here, we report the surprising result that Mmp10 promotes lung tumor initiation and metastasis through the maintenance of a CSC-like population of lung tumor cells.

CSCs are defined by their ability to clonally expand, differentiate into bulk tumor cells, and initiate tumors in recipient animals. Here we show that Mmp10 is overexpressed in CSC-enriched oncosphere cultures from two mouse lung adenocarcinoma cell lines and is required for enhanced transformed growth and clonal expansion *in vitro*, and for tumor initiation *in vivo*. Inhibition of *Mmp10* expression in oncospheres significantly decreases the expression of stem cell-associated transcription factors and cell surface markers, indicating that Mmp10 is critical for maintaining lung CSC identity. Mmp10 expression is also elevated in CSCs isolated from human small cell lung cancer cell lines [27], suggesting that Mmp10 may also function in the maintenance of these CSCs.

A key to effectively targeting NSCLC CSCs will be to accurately identify this subpopulation within tumors. BASCs are the putative tumor-initiating cells from which *Kras*-induced mouse lung tumors originate. BASCs exhibit surface expression of Sca1, Spc and Ccsp, however to date, human bronchioalveolar stem cells that co-express SP-C and CCSP have not been isolated, and Sca1 is not expressed in human lung cells [28]. Interestingly, CMT167 oncosphere cultures are not only enriched in CCSP^+^/SPC^+^ cells (data not shown), but also in CD133^+^/Notch4^+^ cells, two markers implicated in both human and mouse CSCs from various tumor types including lung [25], [29]. Inhibition of Notch signaling blocks the ability of glioblastoma-derived CSC neurospheres to propagate tumors and depletes CD133^+^ stem-like cells in neurospheres [29]. Likewise, Notch inhibition blocks proliferation and tumor-initiating activity of human lung CSCs [25]. We observed significant enrichment of Notch4^+^/CD133^+^ cells in CMT167 oncospheres with few single positive cells suggesting that co-expression of these molecules is required to maintain CMT167 CSCs. Thus, Notch4 and CD133 may be useful markers of lung CSCs across species.

Our data provide direct evidence that the role of *Mmp10* in lung CSCs is cell autonomous. Our orthotopic tumor model shows Mmp10 staining in tumor cells but not tumor-associated stroma or morphologically normal lung epithelium. More importantly, no significant differences were observed in tumor number, size or metastatic lesions formed by oncosphere cells orthotopically injected into syngeneic Ntg or Mmp10^−/−^ mice. Thus, while many Mmps produced by tumor-associated stroma play prominent roles in the invasive and metastatic properties of lung tumor cells, Mmp10 is produced primarily by lung tumor cells to support the autonomous growth of CSCs. Our data are consistent with numerous reports that Mmp10 is expressed in human NSCLC cells and not surrounding normal or tumor-associated lung tissues [15], [17], [18]. Future studies will focus on determining the molecular mechanisms that contribute to Mmp10-mediated CSC proliferation.

Poor outcome in lung cancer patients is due to metastatic dissemination and resistance of tumor cells to chemotherapy, two characteristics ascribed to CSCs. In primary oncosphere-derived tumors we observed enhanced Mmp10 and Notch4 staining at the interface between the tumor and surrounding tissue, suggesting a role for Mmp10^+^/Notch4^+^ cells in tumor invasion. These data are consistent with our previous funding that Mmp10 is required for invasion of human NSCLC cells *in vitro* [19], and our current finding that Mmp10 contributes to the metastatic behavior of CMT167 oncosphere-derived tumors. Interestingly, tumor cells within metastatic lesions uniformly express Mmp10 and Notch4 suggesting that they are derived from, and highly enriched in, CSCs. Mmp10 knockdown leads to a significant reduction in metastatic lesions indicating the importance of Mmp10 in metastatic behavior.

Analysis of expression profiling data on NSCLC revealed a significant correlation between Mmp10 expression and tumor progression, local invasiveness and distant metastasis [30]. We observed similar associations between Mmp10 expression and metastatic behavior of human colorectal cancer, melanoma, breast, renal and prostate cancers (**Table 1**). Thus, Mmp10 may play a widespread role in the metastatic behavior of many types of human cancers. Using a bioinformatics approach, we recently demonstrated that Mmp10 expression in human tumors is associated with both embryonic stem cell gene signatures and metastatic potential [21]. Interestingly, CSCs appear to promote chemoresistance and a recent study revealed that Mmp10 is overexpressed in cisplatin-resistant compared to cisplatin-sensitive human ovarian adenocarcinoma cells [31]. Further studies will be required to determine whether Mmp10 promotes metastasis and chemoresistance in human tumor cells by maintaining a highly metastatic, chemoresistant cancer stem cell population, as our present data in mouse lung adenocarcinoma cells would predict.

## Materials and Methods

### Cell lines, enzymes, and lentiviral RNAi-mediated gene knockdown

CMT167 and LLC cells expressing firefly luciferase were a gift from Dr. Raphael Nemenoff (University of Colorado, Denver, CO) and were characterized previously [32]. Briefly, these cells were transfected with pGL3 containing firefly luciferase constitutively driven by an SV40 promoter and cells expressing high luciferase were selected using neomycin resistance [32]. Recombinant human proMmp10 catalytic domain was expressed in E. coli strain BL21 (DE3), isolated from inclusion bodies, purified, refolded, and activated *in vitro* by treatment with 4-aminophenylmercuric acetate, essentially as described previously for Mmp3 [33]. Details of the construct and methods have been submitted for publication elsewhere [34]. Concentrations of activated Mmp10 were determined by UV absorbance at 280 nm, using a calculated molar extinction coefficient of 29910 M^−1^cm^−1^. Specific activity of Mmp10 (43.75 U/μg; 1 U = 100 pmol/min at 37°C) was determined as described previously [19]. 100 U/ml of Mmp10 was added as indicated in the figure legends.

Lentiviral vectors containing short hairpin RNAi against mouse Mmp10 were obtained from Sigma-Aldrich. A non-target control lentiviral vector containing a short hairpin RNA construct that does not recognize any mouse or human genes (NT) was used as a negative control. Cells were transduced with recombinant lentivirus and stable transfectants selected by puromycin resistance as described previously [19]. RNAi sequences are provided in **Figure S1A**. Efficiency of Mmp10 knockdown was assessed by quantitative PCR (QPCR).

### Enrichment, clonal expansion and redifferentiation of lung CSCs

CSCs were enriched from CMT167 and LLC cells by culturing 10,000 cells/ml in serum-free DMEM-F12 medium (Gibco-Invitrogen, Carlsbad, CA) containing 50 μg/ml insulin (Sigma-Aldrich), 0.4% Albumin Bovine Fraction V (Sigma-Aldrich), N-2 Plus Media Supplement (R&D Systems, Minneapolis, MN), B-27 Supplement (Gibco-Invitrogen), 20 μg/ml EGF (PeproTech) and 10 μg/ml bFGF (PeproTech) (CSC medium) in ultra-low attachment flasks (Corning, Corning, NY) to support growth of undifferentiated oncospheres. Oncosphere cultures were expanded by trypsinization and mechanical dissociation followed by re-plating of single cell suspensions (10,000 cells /ml) in fresh CSC medium. Oncospheres were collected for experiments after 2 weeks in non-adherent culture. For clonal expansion, single cells were added to each well of 96-well ultra-low attachment tissue culture plates (Corning) and clonal expansion was monitored at the indicated time points. Oncosphere diameters were determined using Image-Pro Plus 6.3 (Media Cybernetics, Bethesda, MD). To redifferentiate oncospheres, single cell suspensions were plated in normal growth media (10,000 cells/ml) in dishes supporting cell attachment. Cellular proliferation was assessed by a standard MTT assay using protocols supplied by the manufacturer (Promega Madison, WI).

### RNA isolation and quantitative PCR

Total RNA was extracted from CMT167 and LLC cells using the RNeasy Plus Mini Kit (Qiagen, Valencia, CA). QPCR reagents for mouse Nanog, Oct3/4, Aldh1a7, Notch1, Notch2, Notch3, Notch4, Cd133, Bmi, Hes1, Hes2, Hey1, Hey2, Mmp 2, 7, 9, 10, 11, 12 and 14 mRNA were purchased from Applied Biosystems (Foster City, CA). QPCR was carried out using an Applied Biosystems 7900 thermal cycler, and data was analyzed using the SDS 2.3 software package. Data were normalized to 18S RNA.

### Measurement of Mouse Mmp10 Protein by ELISA

Mmp10 protein in conditioned medium from CMT167 adherent or oncosphere cultures was assessed by ELISA (USCN Life Science Inc., China). Briefly, cells were cultured for 24 hours in fresh media prior to assay. Media were collected, separated by centrifugation and cell number and volume of medium determined. ELISA was performed according to the manufacturer's instructions and results recorded at 450 nm using a SpectraMax M5 (Molecular Devices, Sunnyvale, CA). Results are presented as mean Mmp10 concentration per 1×10^6^ cells +/− SD. Each determination represents triplicate cultures and the results are representative of three independent experiments.

### Flow cytometry

CMT167 adherent and oncosphere cultures were disassociated into single cells by trypsinization followed by filtration through a 40 μm cell strainer. Cells were incubated for 1 hour at 4^°^C with Alexa Fluor 488-conjugated CD133 (Millipore, Billerica, MA) and Alexa Fluor 647 conjugated Notch4 (Biolegend, San Diego, CA) antibodies or respective isotype controls. Cells were incubated for 1 hour at 4^°^C with CCSP (Upstate, Temecula, CA) and SPC (Santa Cruz Biotechnology, Santa Cruz, CA) antibodies followed by a 30 minute incubation with Alexa Fluor 488– and Alexa Fluor 647 conjugated secondary antibodies (Invitrogen, Carlsbad, California). Flow cytometry was performed on an Accuri C6 flow cytometer and analyzed using CFlow Plus software (Accuri Cytometers, Inc., Ann Arbor, MI).

### Anchorage-independent growth and orthotopic implantation studies

Anchorage-independent growth was assessed by seeding CMT167 (1,000 cells/dish) or LLC (500 cells/dish) cells in soft agar on 35 mm culture dishes and quantified after four weeks in culture as described previously [35]. CMT167/luc cell transfectants suspended in 10% Growth Factor Reduced Matrigel Matrix (BD Biosciences) in PBS were injected orthotopically into the left lobe of the lungs of syngeneic C57BL/6 mice using a 30-gauge needle. For bioluminescence imaging, mice were injected intraperitoneally with 150 mg/kg body weight luciferin prior to sedation and imaged using the IVIS Imaging System (Caliper Life Sciences-Xenogen, Hopkinton, MA). Biolumnescent signals were quantified using Living Image software (Caliper Life Sciences-Xenogen). Mouse lung tissues were prepared for histology and immunohistochemistry as previously described [20], [36]. Metastases to the right lobes of the lung were quantitated from hematoxylin/eosin-stained sections. Sections were stained for Mmp10 (NBP1-03118; Novus Biologicals, Littleton, CO) and Notch4 (Santa Cruz, Santa Cruz, CA) and antigen visualized using the Envision Plus Dual Labeled Polymer Kit (DAKO). Images were analyzed using the ScanScope scanner and ImageScope software (Aperio Technologies, Vista, CA). Primary orthotopic tumors were dissected using a dissecting scope and lung epithelial tumor cells were isolated for CD133/Notch4 flow cytometric analysis using a published protocol [37]. Briefly, red blood cells (RBC) were lysed in RBC lysis buffer (StemCell Technologies) and CD45^pos^ and Pecam^pos^ cells were removed using primary biotinylated antibodies to CD45 (BD PharMingen) and Pecam (BD PharMingen) and the EasySep immunomagnetic cell selection procedure (Stem Cell Technologies). FACS analysis was carried out as described above. All animal experiments were approved by the Institutional Animal Care and Use Committee of Mayo Clinic (protocol #A10811).

### Assessment of Mmp10 in primary human cancer datasets

The correlation between Mmp10 expression and various types of human cancer was determined using the NextBio data mining framework (www.nextbio.com) [38]. The degree of correlation calculated by NextBio was based on Mmp10 values for individual microarray studies of specific cancer types as listed in **Table 1**. From these experiments, p-values and fold change measurements, provided by NextBio, were reported.

### Statistical analysis

The Student's *t* test and one-way ANOVA statistical analyses were done using SigmaStat 3.5. A *P* value of less than 0.05 was considered statistically significant.

## Supporting Information

Figure S1 **Characterization of LLC oncospheres.** **A**) Phase contrast photomicrographs showing morphology of parental adherent LLC cells (***left panel***), LLC cells grown as oncospheres in stem cell culture (***middle panel***) and redifferentiated oncosphere cells after return to adherent culture (***right panel***). **B**) LLC oncosphere cultures exhibit enhanced anchorage-independent growth. Mean fold-change from LLC parental cells +/−SEM. n = 5, *p<0.00001; **p<0.00002. **C**) QPCR for stem cell markers in parental, oncosphere and redifferentiated oncosphere cultures. Fold of parental cells +/−SEM, n = 3; *p<0.05.
(TIF)

Figure S2 **Characterization of** **mouse lentiviral Mmp10 RNAi constructs**. Lentiviral shRNA constructs targeting mouse MMP-10 were obtained from Sigma Misssion RNAi. **A**) Listed are the assigned construct #, and the target sequences of each RNAi construct. All constructs targeted the coding sequence (CDS) of the Mmp10 mRNA. An asterisk indicates the construct giving the most efficient knock down of the Mmp10 mRNA, which was used in the experiments described in the text. **B**) QPCR analysis of Mmp10 mRNA abundance in NT and Mmp10 RNAi CMT167 cells. CMT167 cells were stably transduced with one of three lentiviruses expressing an RNAi construct targeting Mmp10 or a non-target control lentivirus as described in ***Materials and Methods***. Cells were harvested and analyzed by QPCR for Mmp10 mRNA abundance. Results are expressed as % NT control. Columns, mean; bars, SEM, n = 3, (*) denotes p<0.05 relative to NT control. **C**) Effect of Mmp10-RNAi constructs on anchorage-independent growth in soft agar. Results are expressed as % NT control. Columns, mean; bars, SEM, n = 5, (*) denotes p<0.05 relative to NT control. Construct #3 was used in subsequent experiments.
(TIF)

Figure S3 **Mmp10 is critical for maintenance and growth of LLC CSC cultures**. **A**) QPCR showing RNAi-mediated knockdown of Mmp10 in parental LLC and oncosphere cultures. Relative to NT parental cell control; Mean ±s.d., n = 3. *p<0.00003 and **p<0.000001 vs. NT parental control. **B**) Effect of Mmp10 RNAi on anchorage-independent growth of parental and LLC oncosphere cultures. Relative to NT Parental cells +/− SEM, n = 5; *p<0.05 vs, NT; **p<0.05 vs. NT parental. **C**) QPCR for stem cell markers in NT parental, and NT and Mmp10 RNAi LLC oncosphere cells. Fold of NT parental cells +/−SEM, n = 3; *p<0.05. **D**) Colony diameter of oncospheres derived from single LLC NT and Mmp10 RNAi CSCs. Mean +/− SEM; n = 10, *p<0.05 vs. day 1 each treatment; **p<0.05 vs. day 8 NT RNAi.
(TIF)

Figure S4 **Mmp10 is required for stem cell gene expression in CMT167 oncosphere cultures**. **A**) QPCR analysis of genes associated with stemness in CMT167 NT oncosphere cultures, and untreated or recombinant Mmp10-treated Mmp10 RNAi oncosphere cells. Results are expressed as % NT oncosphere cultures. **B**) Mmp10-deficient CMT167 oncosphere cultures exhibit normal cell proliferation upon redifferentiation. NT and Mmp10 RNAi CMT167 oncosphere cultures were placed into adherent culture in the presence of serum to induce differentiation. Cells were assessed for growth rate by MTT assay as described in ***Materials and Methods***. Results represent the mean +/− S.D.; n = 5.
(TIF)

Figure S5 **Mmp10 dependent tumorigenic and metastatic activity of orthotopic CMT167 tumors.** **A**) Orthotopic tumor growth was monitored by bioluminescence at the indicated time points after injection of 100,000 NT or Mmp10 RNAi adherent CMT167 cells *p<0.05, n = 10. **B**) Metastases in NT RNAi adherent, and NT and Mmp10 RNAi oncosphere tumors. *p<0.02 and **p<0.03 vs. NT adherent cell tumors.
(TIF)
