# Supplementary figures and images for: Matrix Metalloproteinase-10 Is Required for Lung Cancer Stem Cell Maintenance, Tumor Initiation and Metastatic Potential
Source: PLoS One. 2012 Apr 24;7(4):e35040. doi: 10.1371/journal.pone.0035040 (PMC3335833; doi:10.1371/journal.pone.0035040)

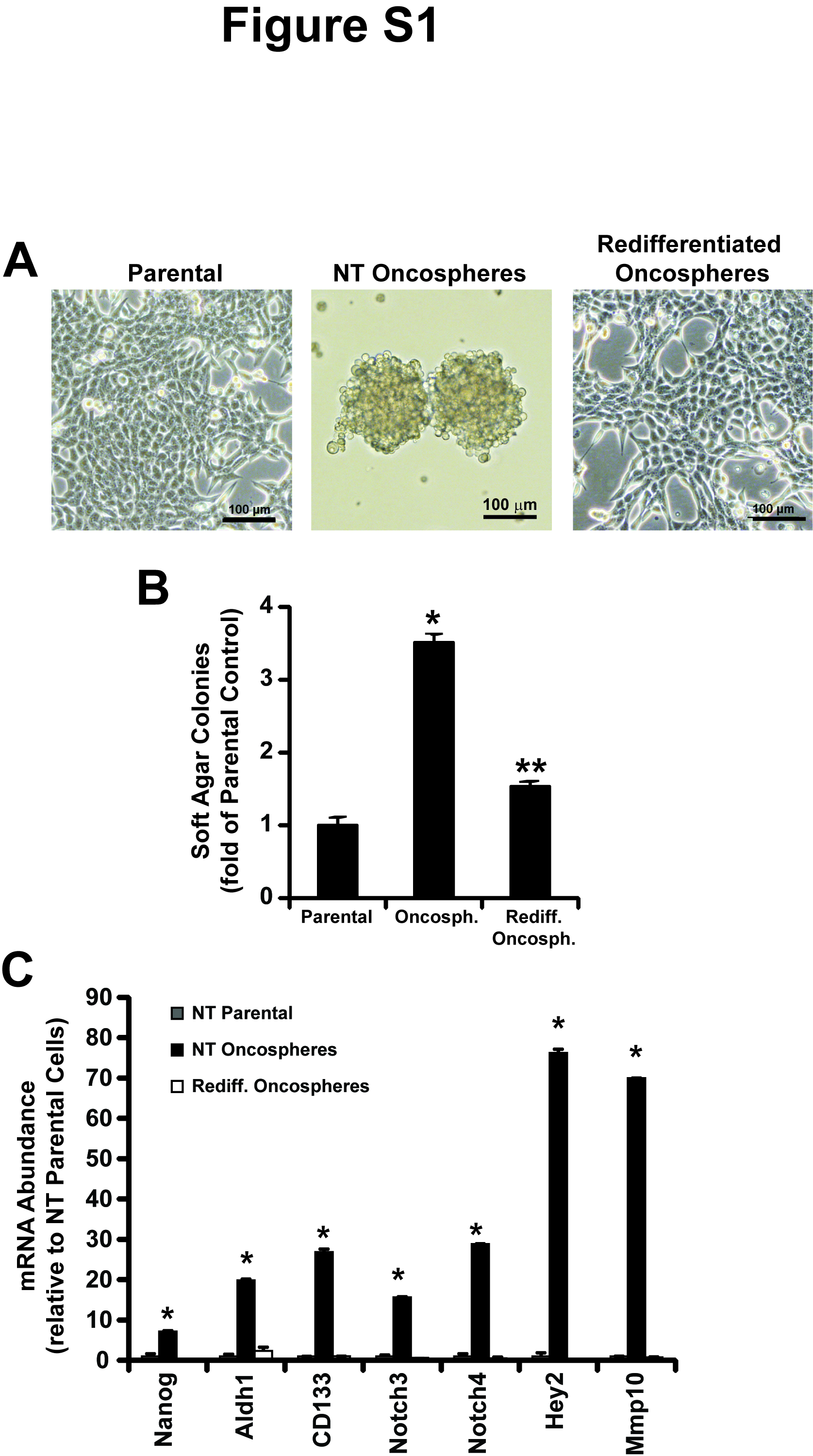

Supplement: Figure S1 — Characterization of LLC oncospheres. A) Phase contrast photomicrographs showing morphology of parental adherent LLC cells (left panel), LLC cells grown as oncospheres in stem cell culture (middle panel) and redifferentiated oncosphere cells after return to adherent culture (right panel). B) LLC oncosphere cultures exhibit enhanced anchorage-independent growth. Mean fold-change from LLC parental cells +/−SEM. n = 5, *p<0.00001; **p<0.00002. C) QPCR for stem cell markers in parental, oncosphere and redifferentiated oncosphere cultures. Fold of parental cells +/−SEM, n = 3; *p<0.05. (TIF) [file pone.0035040.s001.tif]

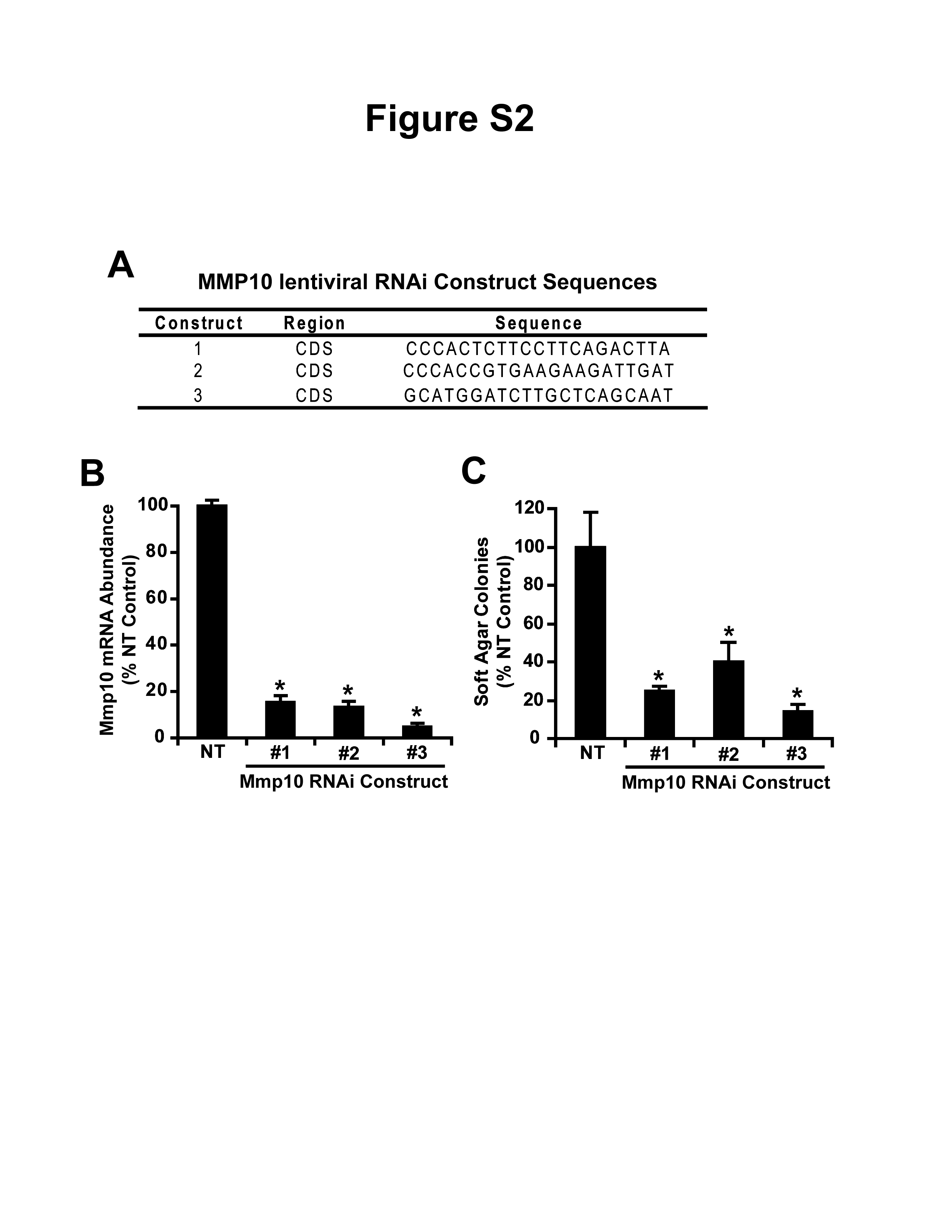

Supplement: Figure S2 — Characterization of mouse lentiviral Mmp10 RNAi constructs. Lentiviral shRNA constructs targeting mouse MMP-10 were obtained from Sigma Misssion RNAi. A) Listed are the assigned construct #, and the target sequences of each RNAi construct. All constructs targeted the coding sequence (CDS) of the Mmp10 mRNA. An asterisk indicates the construct giving the most efficient knock down of the Mmp10 mRNA, which was used in the experiments described in the text. B) QPCR analysis of Mmp10 mRNA abundance in NT and Mmp10 RNAi CMT167 cells. CMT167 cells were stably transduced with one of three lentiviruses expressing an RNAi construct targeting Mmp10 or a non-target control lentivirus as described in Materials and Methods. Cells were harvested and analyzed by QPCR for Mmp10 mRNA abundance. Results are expressed as % NT control. Columns, mean; bars, SEM, n = 3, (*) denotes p<0.05 relative to NT control. C) Effect of Mmp10-RNAi constructs on anchorage-independent growth in soft agar. Results are expressed as % NT control. Columns, mean; bars, SEM, n = 5, (*) denotes p<0.05 relative to NT control. Construct #3 was used in subsequent experiments. (TIF) [file pone.0035040.s002.tif]

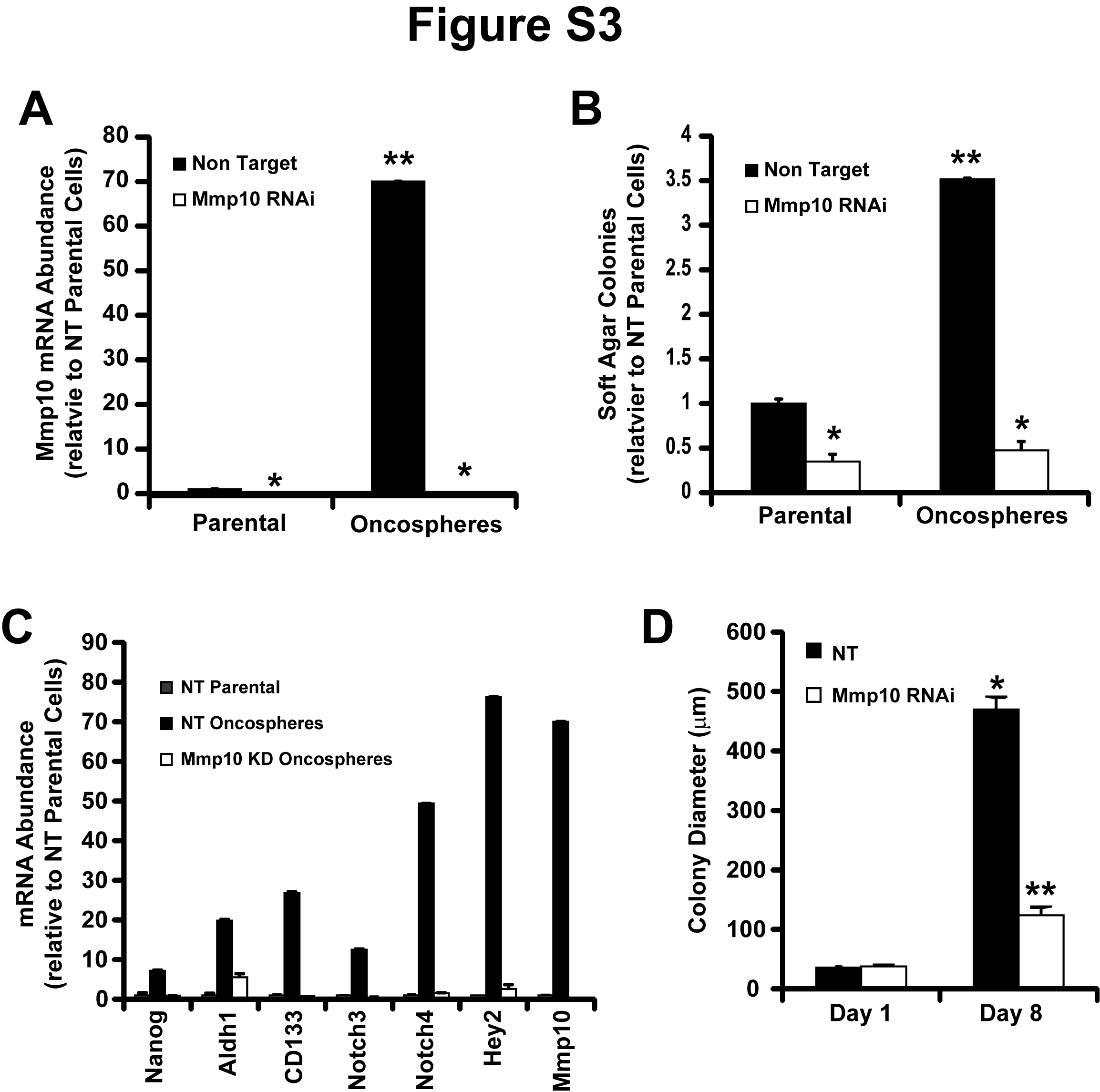

Supplement: Figure S3 — Mmp10 is critical for maintenance and growth of LLC CSC cultures. A) QPCR showing RNAi-mediated knockdown of Mmp10 in parental LLC and oncosphere cultures. Relative to NT parental cell control; Mean ±s.d., n = 3. *p<0.00003 and **p<0.000001 vs. NT parental control. B) Effect of Mmp10 RNAi on anchorage-independent growth of parental and LLC oncosphere cultures. Relative to NT Parental cells +/− SEM, n = 5; *p<0.05 vs, NT; **p<0.05 vs. NT parental. C) QPCR for stem cell markers in NT parental, and NT and Mmp10 RNAi LLC oncosphere cells. Fold of NT parental cells +/−SEM, n = 3; *p<0.05. D) Colony diameter of oncospheres derived from single LLC NT and Mmp10 RNAi CSCs. Mean +/− SEM; n = 10, *p<0.05 vs. day 1 each treatment; **p<0.05 vs. day 8 NT RNAi. (TIF) [file pone.0035040.s003.tif]

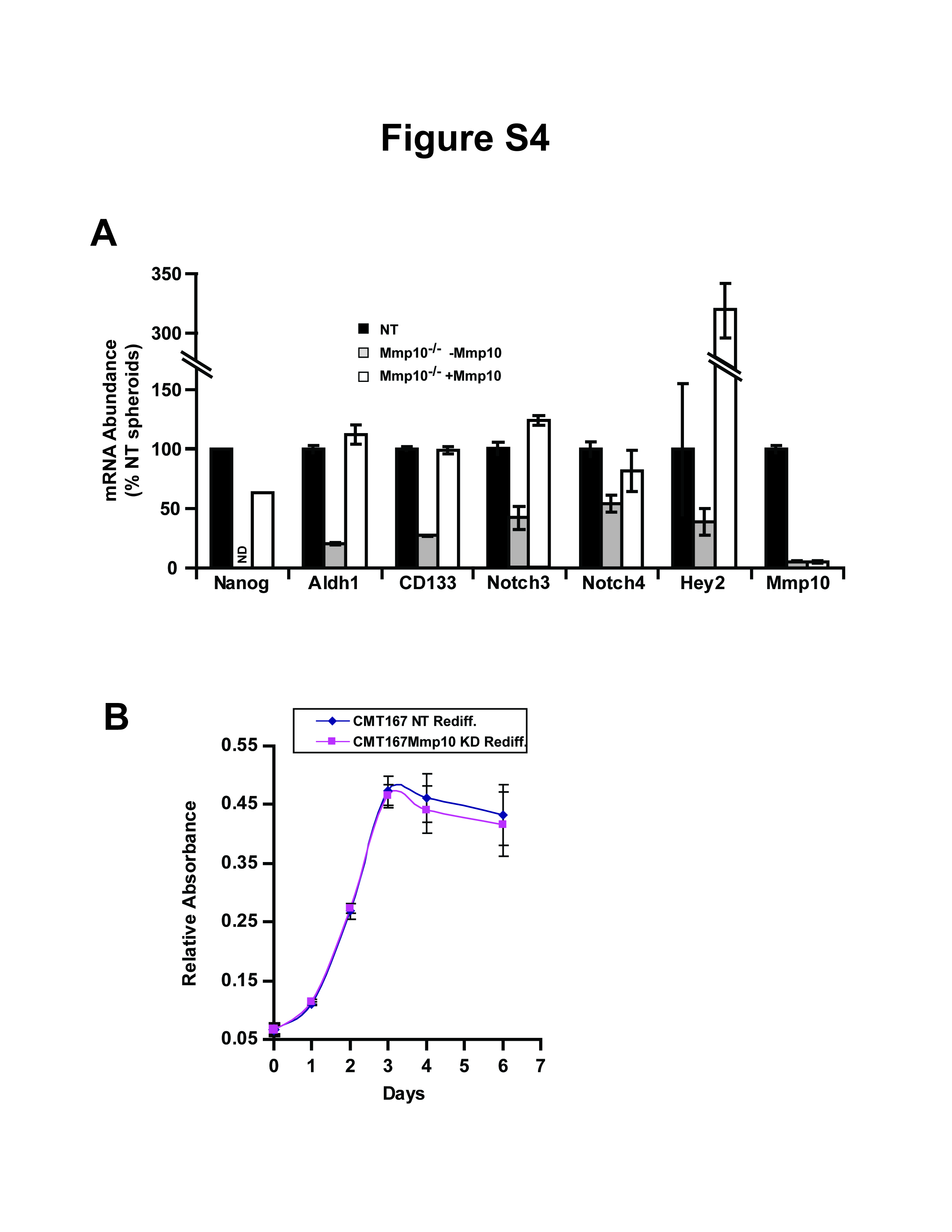

Supplement: Figure S4 — Mmp10 is required for stem cell gene expression in CMT167 oncosphere cultures. A) QPCR analysis of genes associated with stemness in CMT167 NT oncosphere cultures, and untreated or recombinant Mmp10-treated Mmp10 RNAi oncosphere cells. Results are expressed as % NT oncosphere cultures. B) Mmp10-deficient CMT167 oncosphere cultures exhibit normal cell proliferation upon redifferentiation. NT and Mmp10 RNAi CMT167 oncosphere cultures were placed into adherent culture in the presence of serum to induce differentiation. Cells were assessed for growth rate by MTT assay as described in Materials and Methods. Results represent the mean +/− S.D.; n = 5. (TIF) [file pone.0035040.s004.tif]

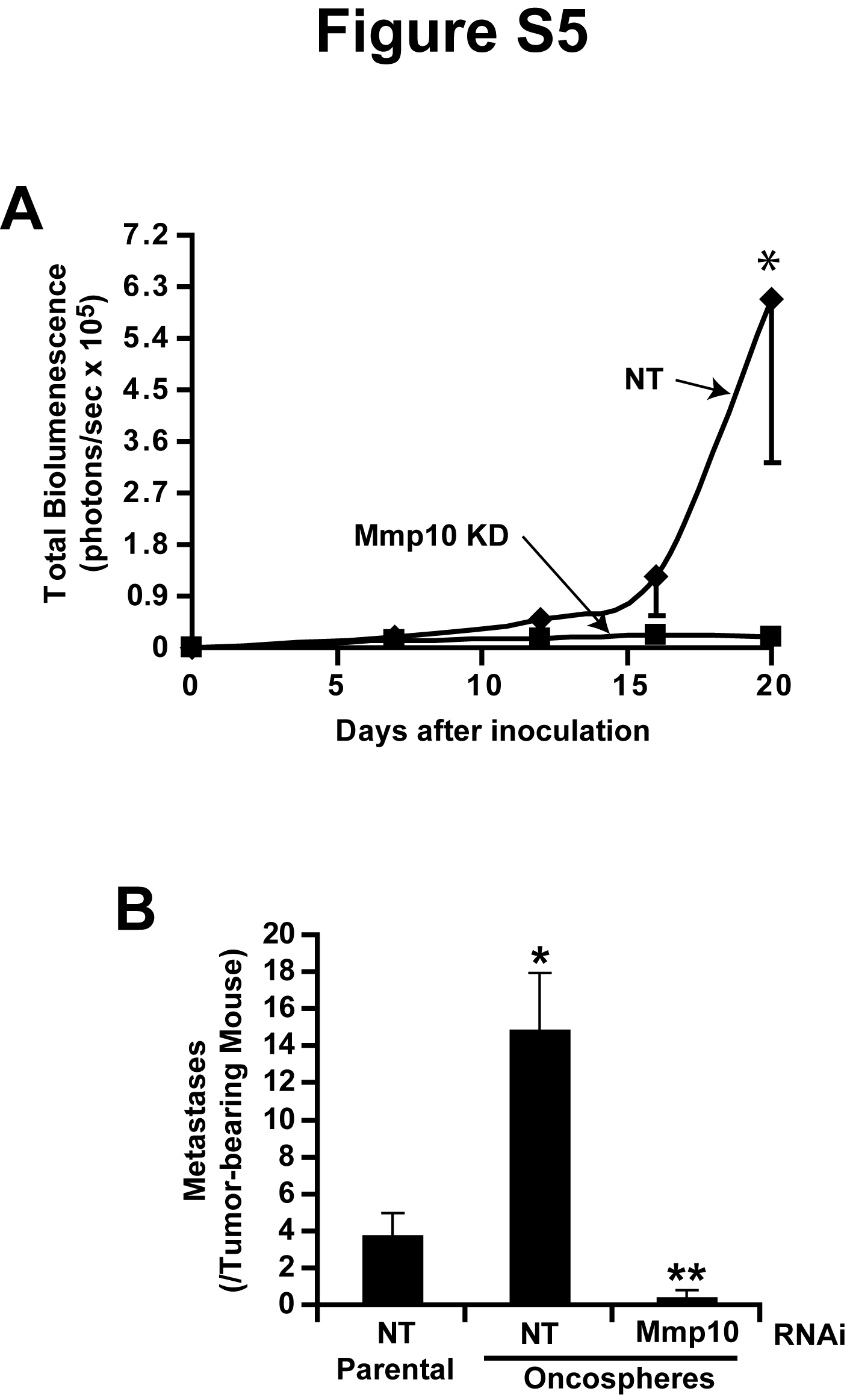

Supplement: Figure S5 — Mmp10 dependent tumorigenic and metastatic activity of orthotopic CMT167 tumors. A) Orthotopic tumor growth was monitored by bioluminescence at the indicated time points after injection of 100,000 NT or Mmp10 RNAi adherent CMT167 cells *p<0.05, n = 10. B) Metastases in NT RNAi adherent, and NT and Mmp10 RNAi oncosphere tumors. *p<0.02 and **p<0.03 vs. NT adherent cell tumors. (TIF) [file pone.0035040.s005.tif]
